# 2484: Establishment of the Tennessee-sickle cell disease network as a mechanism for engaging a rare disease population in patient centered outcomes research

**DOI:** 10.1017/cts.2017.284

**Published:** 2018-05-10

**Authors:** Tilicia Mayo-Gamble, Velma McBride Murry, Michael R. DeBaun

**Affiliations:** Vanderbilt University, Nashville, TN, USA

## Abstract

OBJECTIVES/SPECIFIC AIMS: Despite the high prevalence of individuals diagnosed with sickle cell disease (SCD) in Tennessee, comprehensive care and education for patients with SCD is not as widely available as healthcare services for individuals managing other chronic illnesses. We aimed to engage SCD stakeholders in patient-centered outcomes research (PCOR) as a mechanism for advancing care and translational research for this rare disease population. METHODS/STUDY POPULATION: Through a partnership with the Sickle Cell Foundation of Tennessee, we implemented Community Health Ambassadors to systematically engage patient partners with SCD and their caregivers, aged 18–50 from rural and urban communities throughout Tennessee, in PCOR to establish a sustainable infrastructure, focused on connecting the SCD community through a service providing community-based organization to offer (1) information on how to connect with other families; and be informed about SCD community activities, or educational offerings; (2) training in basic research principals; and (3) opportunities to contribute to PCOR, including feedback on effective and practical ways for providing input on research efforts through patient centered input, comparing urban and rural area preferences. Community ambassadors utilized health fairs, clinic days at various hospitals and community centers, and social media to spread awareness of the project, in addition to boosting the recruitment process. RESULTS/ANTICIPATED RESULTS: A statewide SCD network was developed to offer social support and increase access to education, medical care, and engagement in research activities. Findings include: recruitment of 150 patients and 35 executive committee members (local physicians, community leaders, adults with SCD and parents of children with SCD). DISCUSSION/SIGNIFICANCE OF IMPACT: Most rural and urban families affected by SCD have no systematic way to engage in, or lend their expertise to, PCOR. A statewide network of patient partners, community stakeholders, researchers, and medical professionals will ultimately increase the standard of care for patients, and provide valuable insight for SCD research. The opportunity to create the underpinnings for coordinated patient-centered education for patients with SCD and their caregivers holds promise for developing a scalable PCOR process model for replication and implementation in other states and emulate this model with other rare disease populations.

